# Zbtb38 is a novel target for spinal cord injury

**DOI:** 10.18632/oncotarget.17487

**Published:** 2017-04-27

**Authors:** Yafei Cai, Jun Li, Zongmeng Zhang, Jing Chen, Yangzi Zhu, Rui Li, Jie Chen, Lixia Gao, Rong Liu, Yong Teng

**Affiliations:** ^1^ College of Animal Science and Technology, Nanjing Agricultural University, Nanjing 210095, China; ^2^ College of Life Sciences, Anhui Normal University, Wuhu 241000, China; ^3^ Department of Biochemistry and Molecular Biology, Augusta University, Augusta GA 30912, USA; ^4^ Department of Oral Biology, Augusta University, Augusta GA 30912, USA; ^5^ College of Food Science and Technology, Nanjing Agricultural University, Nanjing 210095, China

**Keywords:** Zbtb38, spinal cord injury, ATF4, ER stress, apoptosis

## Abstract

Spinal cord injury (SCI) is currently incurable since treatments applied to clinic are limited to minimizing secondary complications and the mechanisms of injury-induced spinal cord damage are poorly understood. Zbtb38, also called CIBZ, is highly expressed in spinal cord and it functions as a negative regulator in SCI-induced apoptosis. We show here that Zbtb38 is downregulated under endoplasmic reticulum (ER) stress, which promotes ER stress-associated apoptosis in human bone marrow neuroblastoma cells. In the traumatic SCI mice, ER stress presented in injured spinal cord induced repression of Zbtb38 expression and triggered Zbtb38-mediated apoptosis. ChIP-QPCR analysis revealed that ATF4, an ER-stress inducible transcription factor, directly activated Zbtb38 transcription by binding to the Zbtb38 promoter. However, this binding was significantly reduced following SCI, leading to a sharp decrease in Zbtb38 expression. Restoring Zbtb38 function in injured spinal cord by injection of lentivirus containing Zbtb38 into SCI mice, significantly alleviated secondary damage of spinal cord with decreased ER stress-associated apoptosis and partially recovered spinal cord functions. These findings demonstrate that restoration of Zbtb38 expression can reduce secondary tissue damage after SCI, and suggest that a therapeutic strategy for targeting Zbtb38 may promote functional recovery of spinal cord for patients with SCI.

## INTRODUCTION

Spinal cord injury (SCI) represents damage of the spinal cord resulting from a blunt or penetrating trauma, which is a severe disease of the central nervous system (CNS) disrupting important signals between the nervous system and muscles and creating an imbalance that increases muscle activity or spasms [1–3]. Provision of care for individuals with SCI is usually met with frustration, and actions to treat and rehabilitate following SCI are very limited [4, 5]. Scientists and clinicians have started to take up the challenge of discovering and validating different novel approaches for the treatment of SCI. One possible strategy to speed translation of new interventions into the clinic is the identification of major players of SCI.

SCI induces both primary uncontrollable mechanical injury and secondary controllable degeneration [6]. The activation of cell death cascades following SCI mediates delayed tissue degeneration and contributes to secondary damage of spinal cord[6]. Several reports have suggested that apoptosis is an important contributing factor in the secondary damage in animal models and in human tissue [7, 8]. Therefore, it will be feasible to improve functional outcome of injured spinal cord if blocking SCI-induced apoptosis effectively. In previous study, we have found that Zbtb38/CIBZ, a protein forms homodimers or heterodimers through the zinc finger domains, is implicated in the SCI process [9]. In the traumatic SCI mouse model, Zbtb38 expression was dramatically decreased in spinal cord, which triggered apoptosis through p53-independent Caspase3 pathway, and led to enhanced secondary damage of spinal cord [9]. However, which signal induces repression of Zbtb38 expression in injured spinal cord remains unexplored.

Endoplasmic reticulum (ER) stress can reduce and remove the excessive damage to cells in response to various cellular stresses caused by physiological or pathological stimuli, including those from infection, trauma and oxidative damage [10]. ER stress can induce activation of the unfolded protein response (UPR) and upregulate the expression levels of UPR target genes (*e.g*. Grp78, Chop and ERdj4) [11–13]. In addition, ER stress can promote phosphorylation of the eukaryotic translation initiation factor 2 (eIF2A) through accumulation and aggregation of unfolded proteins [14, 15]. Many studies have demonstrated that persistent or severe ER stress can induce unexpected apoptosis [16–18]. More research is needed to understand the mechanisms of ER stress-associated apoptosis in SCI, although ER stress responses seem to divert towards apoptosis pathways in neurons and oligodendrocytes following SCI in the adult rat [8].

We uncover here that Zbtb38 is a negative regulator of ER stress-associated apoptosis. ER stress blocked the transcription factor ATF4 to bind to the Zbtb38 gene promoter, which in turn inhibited Zbtb38 expression to trigger apoptosis in human bone marrow neuroblastoma cells and injured mouse spinal cord. Restoration of Zbtb38 expression by injecting purified Zbtb38-containing lentivirus into injury center of SCI mice effectively suppressed ER stress-associated apoptosis and rescued spinal cord functions. This study suggests that ATF4-Zbtb38 plays a critical role in SCI-induced apoptosis. Sustaining elevated levels of Zbtb38 in injured spinal cord has the potential to develop a therapeutic strategy for the treatment of SCI patients.

## RESULTS

### ER stress triggers Zbtb38-mediated apoptosis in human bone marrow cells

**Figure 1:**
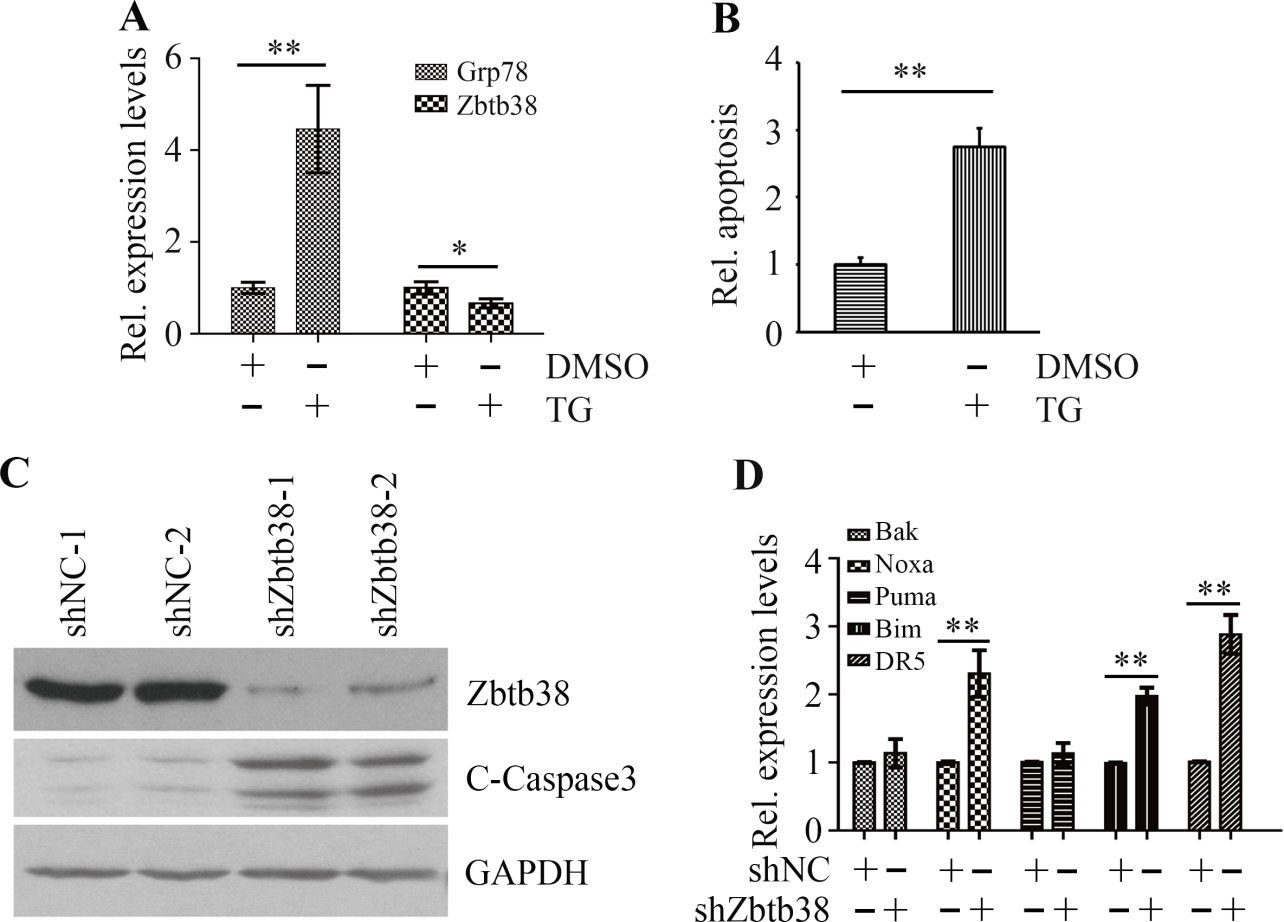
ER stress triggers Zbtb38-mediated apoptosis in SH-SY5Y cells (**A**) SH-SY5Y cells were treated with 1 μM TG for 24 hours and RNA was collected for QRT-PCR analysis. (**B**) Apoptosis of SH-SY5Y cells in the presence or absence of TG was determined by The Cell Death Detection Elisa Kit. (**C**) SH-SY5Y cells were transfected with scramble shRNA (shNC-1 and shNC-2) or shRNA against Zbtb38 (shZbtb38-1 and shZbtb38-2) for 72 hours and cell lysates were collected for Western blot analysis. (**D**) RNA from Zbtb38 knockdown SH-SY5Y cells (shZbtb38) and control cells (shNC) was collected for QRT-PCR analysis to determine the expression levels of pro-apoptotic genes. *p < 0.05; **p < 0.01.

To study whether ER stress affects Zbtb38 expression, we treated human bone marrow neuroblastoma cells SH-SY5Y with 1 μM thapsigargin (TG) and determined Zbtb38 expression by quantitative RT-PCR (QRT-PCR). TG treatment induced ER stress in SH-SY5Y cells as indicated by elevated expression levels of Grp78, an endogenous ER stress marker (Figure 1A). Whereas, a decrease in Zbtb38 expression and a sharp increase in apoptosis of SH-SY5Y cells were observed following TG exposure (Figure 1A and 1B). Zbtb38 is highly expressed in mouse spinal cord and downregulation of Zbtb38 enhances apoptosis following SCI [9, 19]. To investigate the possible function of Zbtb38 in spinal cord, we knocked down Zbtb38 gene by shRNA in SH-SY5Y cells. The levels of cleaved Caspase3 were increased in Zbtb38 knockdown cells compared with knockdown control cells (Figure 1C). We next determined the expression levels of pro-apoptotic genes when Zbtb38 was depleted. Zbtb38 loss led to a significant increase in the expression levels of Noxa, Bim and DR5 genes (Figure 1D). No apparent difference of Bak and Puma expression was identified with or without Zbtb38 loss (Figure 1D). These data indicate that ER stress triggers apoptosis of bone marrow cells, at least in part, by inhibition of Zbtb38.

### Zbtb38 loss induces ER stress-associated apoptosis in the traumatic SCI mice

**Figure 2:**
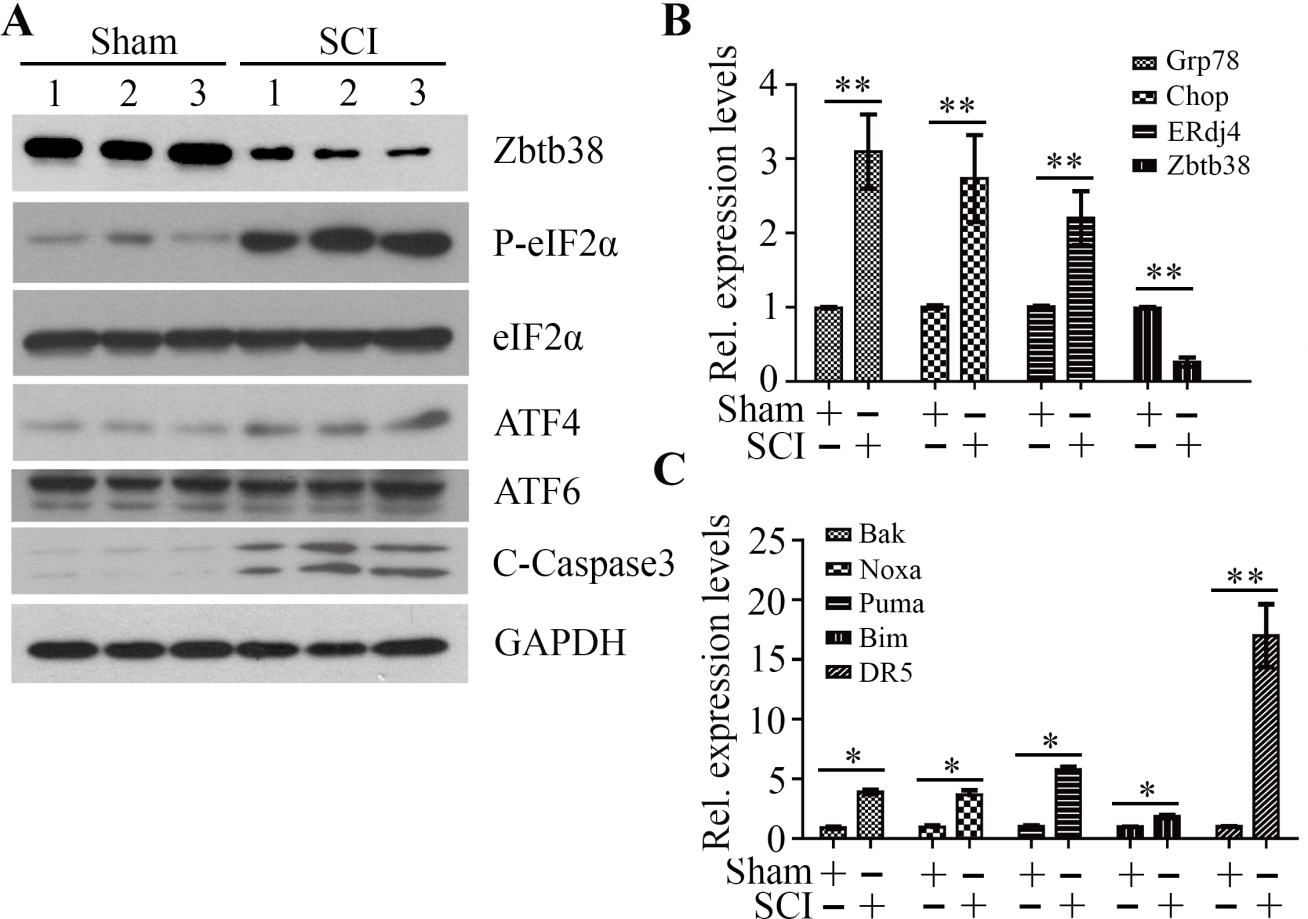
Decreased Zbtb38 expression levels are associated with increased ER stress-associated apoptosis in SCI mice (**A**) Kunming mice were divided into two groups underwent sham injuries (Sham) and SCI, respectively (*n* = 10). The spinal cord samples were harvested from these mice on the day 4 after injury and cell lysates were collected for Western blot analysis. 1, 2, 3 indicate three different mice in each group. (**B, C**) RNA from these samples was collected for QRT-PCR analysis to determine the expression levels of ER stress markers (B) and pro-apoptotic genes (C). *p < 0.05; **p < 0.01.

To investigate the importance of Zbtb38 in the process of SCI, we generated a traumatic SCI mouse model as described previously [9, 20, 21]. We first determined whether SCI induced ER stress in the spinal cord tissues within 4 days after injury. Increased phosphorylation levels of eIF2A and elevated expression levels of several UPR target genes (Grp78, Chop and ERdj4) were found in the traumatic SCI mice (Figure 2A and 2B), which indicate that ER stress is present in the injured spinal cord. We then determined Zbtb38 expression and apoptosis following SCI. Consistent with the observations we reported previously [9], the expression levels of Zbtb38 were significantly decreased after injury, with increased cleaved Caspase3 levels (Figure 2A and 2B). We further determined the expression levels of the pro-apoptotic genes. All the genes examined in this study, including Bak, Noxa, Puma, Bim and DR5, were sharply increased in SCI samples after injury (Figure 2C). Among them, the expression of DR5 was increased almost 17 times in SCI samples compared with non-SCI controls (Figure 2C).

### ATF4 downregulates Zbtb38 expression by reducing the binding amount at the promoter of Zbtb38 gene in traumatic SCI mice

**Figure 3:**
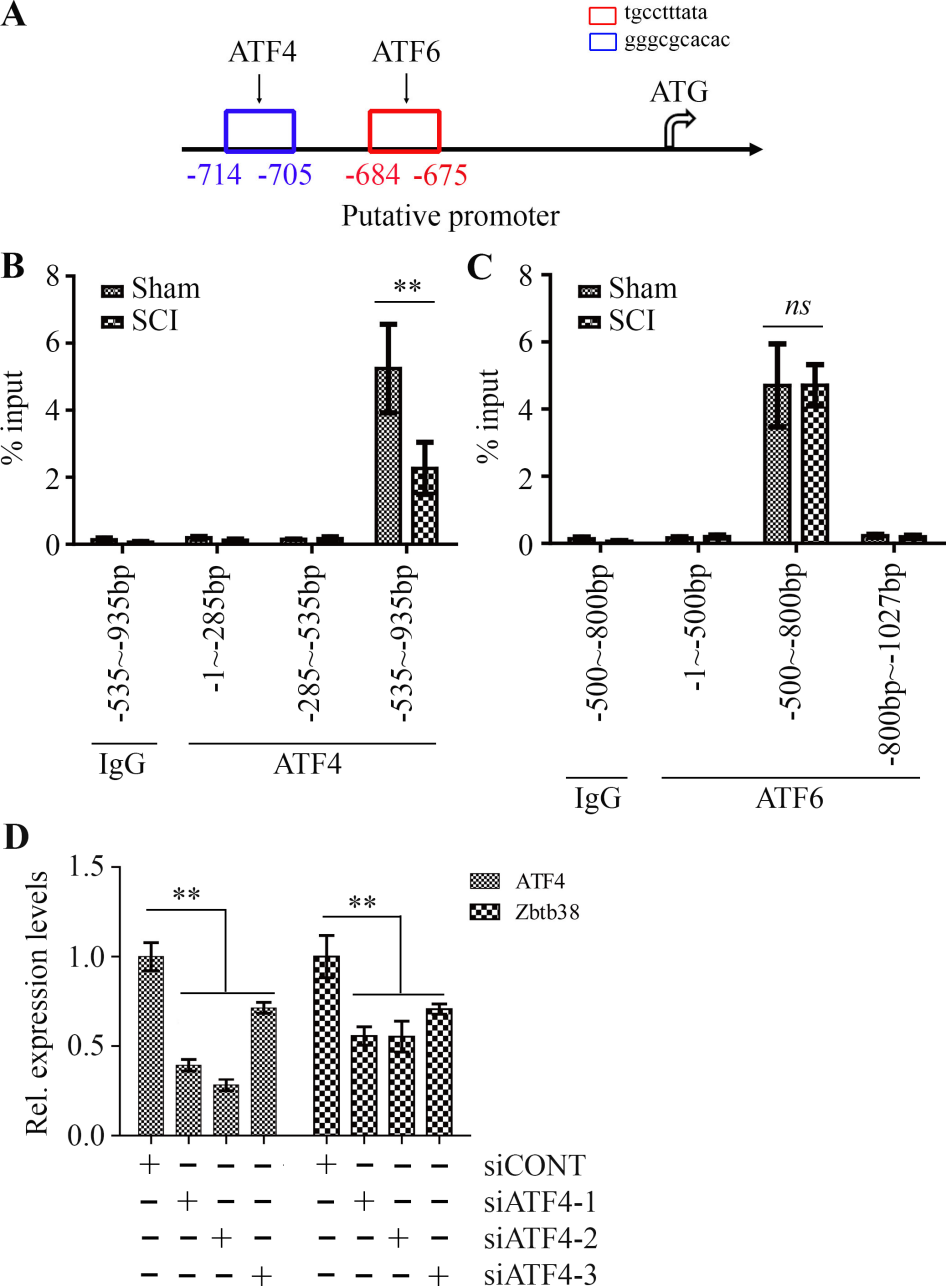
ATF4 regulates Zbtb38 expression in SCI animal model through direct binding (**A**) The cartoon showed the Zbtb38 promoter region upstream of the start codon contains a putative ATF4 and ATF6 binding sites. (**B, C**) The cell lysates from spinal cord samples were pulled down by ATF4 (B) or ATF6 (C) antibody and qPCR primers were used to amplify indicated different regions within the Zbtb38 promoter. The level of enrichment of the indicated DNA sequence was determined relative to the total amount of input DNA (% input). (**D**) SH-SY5Y cells were transfected with scramble siRNA (siCONT) or siRNA against ATF4 (siATF4-1, siATF4-2 and siATF4-3) for 72 hours and RNA was collected from these cells for QRT-PCR analysis. **p < 0.01 and *ns* indicates no significance.

ATF6 and ATF4 are two critical ER stress-inducible transcription factors, leading to specific changes in their target gene expression in response to ER stress [22, 23]. A slight increase in ATF4 expression and no significant change of ATF6 expression were observed after SCI (Figure 2A). Promoter analysis showed ATF4 and ATF6 binding sites within the promoter of Zbtb38 gene (Figure 3A), which prompted us to test whether ATF4 and/or ATF6 bind to the Zbtb38 promoter. To address it, we carried out ChIP assays which showed specific occupancy of ATF4 and ATF6 at the Zbtb38 promoter-binding sites in the spinal cord tissue samples (Figure 3B and 3C). These data suggest that ATF4 and ATF6 are potential transcript factors activate Zbtb38 expression. Intriguingly, decreased levels of ATF4 at the Zbtb38 promoter were seen in SCI samples harvested at 4 days post-injury compared with non-SCI samples (Figure 3B). There was no association of ATF6 occupancy with Zbtb38 expression in spinal cord samples with or without injury (Figure 3C). To confirm that ATF4 regulates Zbtb38, we depleted ATF4 by siRNA in SH-SY5Y cells. Knockdown of ATF4 led to a sharp decrease in Zbtb38 expression (Figure 3D). Taken together, these observations demonstrate that ATF4 acts as a key mediator to promote Zbtb38 expression by binding to the Zbtb38 promoter, and this binding is significantly reduced during SCI.

### ER stress-associated apoptosis is rescued by restoring Zbtb38 expression in traumatic SCI mice

**Figure 4:**
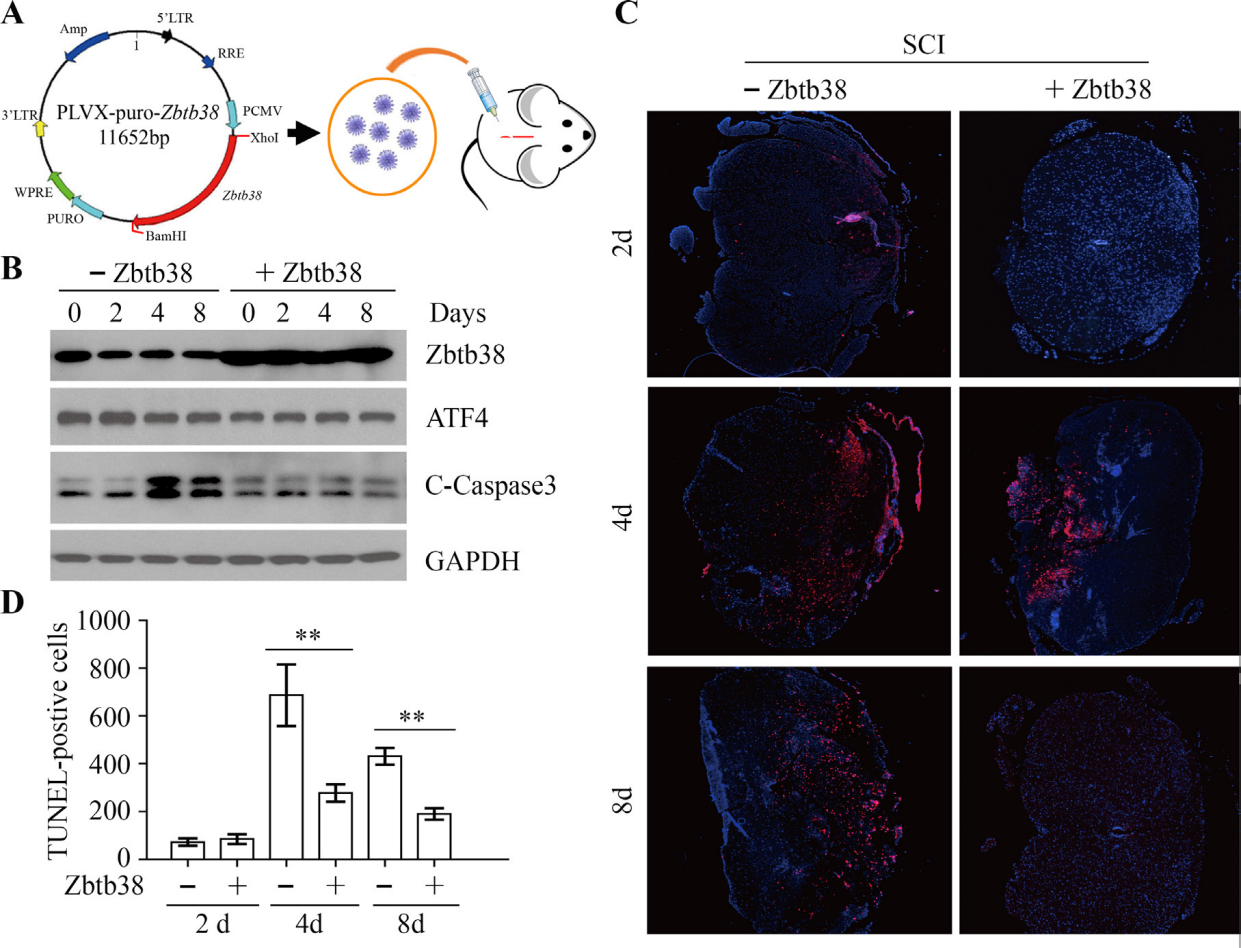
Restoration of Zbtb38 expression rescues SCI-induced apoptosis *in vivo* (**A**) The cartoon showed the flowchart of the injection of lentivirus containing Zbtb38. (**B**) The spinal cord samples were harvested from the SCI mice receiving empty lentivirus (−Zbtb38) or Zbtb38-contaning lentivirus (+Zbtb38) on days 0, 2, 4 and 8 post treatment (*n* = 10), and cell lysates were collected for Western blot. Representative data of this analysis were shown here. (**C, D**) The tissue sections from the same samples were used for TUNEL assays and TUNEL-positive cells were counted. Representative images of these assays are shown in (C) and quantitative data are shown in (D). *p < 0.05; **p < 0.01.

To determine whether restoring Zbtb38 expression could rescue apoptotic death after SCI, we generated a lentiviral construct carrying full-length Zbtb38 cDNA and produced purified lentivirus particle to deliver Zbtb38 into the injury center (Figure 4A). The exogenous Zbtb38 was stably expressed in injured spinal cord within 8 days after injection (Figure 4B) and no toxicity effects were observed in SCI mice when and after injection of Zbtb38-containing lentivirus. Reduced levels of cleaved Caspase3 (Figure 4B and Supplementary Figure 1) and less TUNEL-positive cells (Figure 4C and 4D) were seen when Zbtb38 expression was restored in the injured spinal cord. These findings indicate that upregulation of Zbtb38 can abrogate ER stress-associated apoptosis in injured mouse spinal cord.

**Figure 5:**
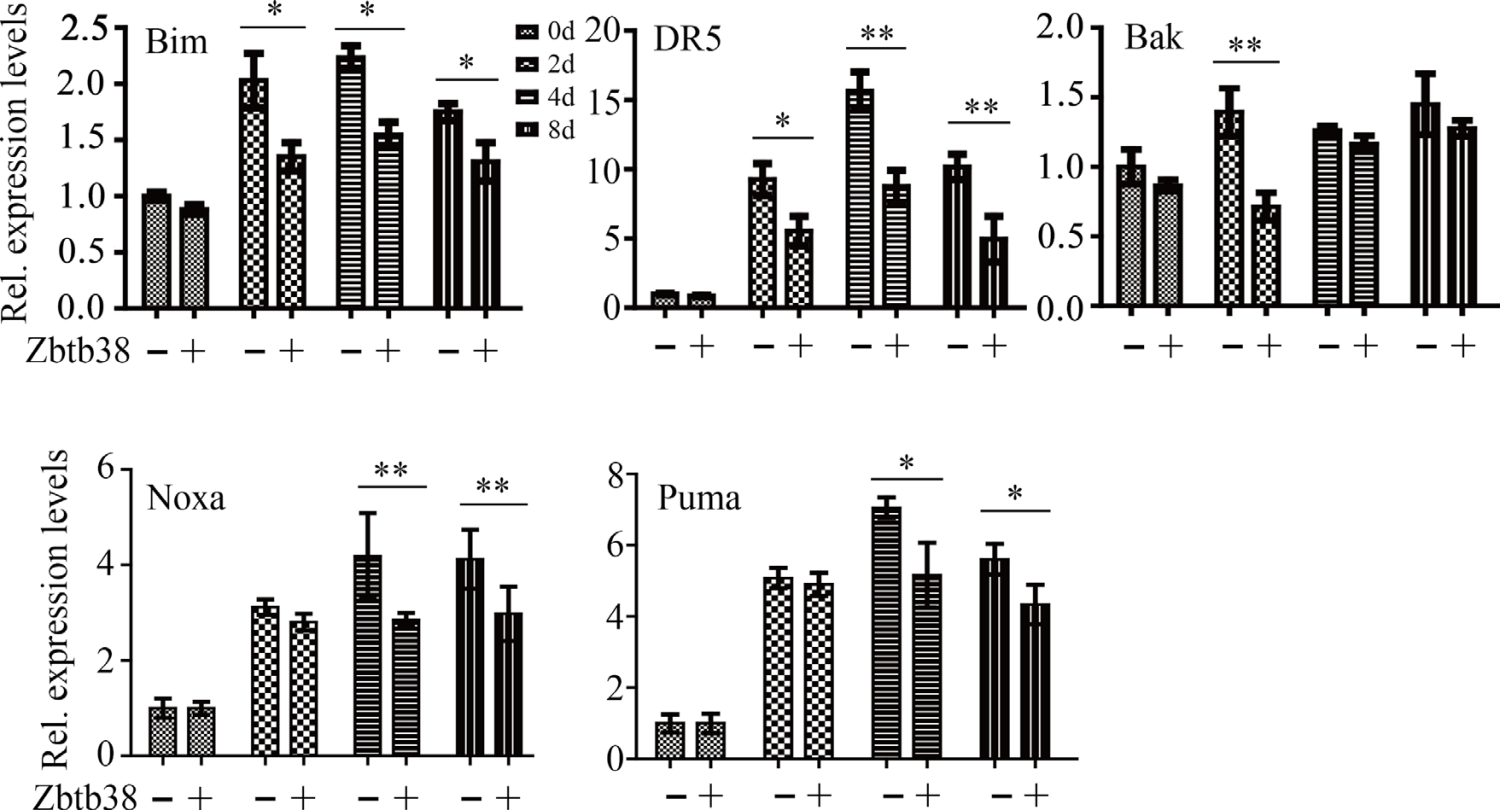
Restoration of Zbtb38 expression attenuates SCI-induced apoptosis *in vivo* RNA was collected from the samples used in Figure 4 for QRT-PCR analysis. *p < 0.05; **p < 0.01.

To further explore the mechanism involved in these events, we examined the expression levels of pro-apoptotic genes, in the presence or absence of the exogenous Zbtb38. The expression levels of Bim and DR5 were significantly lower in the spinal cord samples from Zbtb38 lentivirus-treated SCI mice on the days 2, 4 and 8 of testing, compared with those from empty lentivirus-treated SCI mice (Figure 5). Noxa and Puma, their expression levels were decreased in Zbtb38 lentivirus-treated SCI mice from day 4 after injection, whereas Bak expression was only significantly lower on the days 2 of testing when Zbtb38 expression was rescued (Figure 5). Zbtb38 has been implicated to promote proliferation and G1/S transition in mouse embryonic stem cells [24]. We next determined cell cycle with or without Zbtb38 lentivirus administration. Restoration of Zbtb38 not only inhibited SCI-induced apoptosis but also promoted the spinal cord cell transitions from G1 to S phase (Supplementary Figure 2), confirming that Zbtb38 loss triggers apoptosis and induces suppression of cell proliferation in SCI.

### Restoration of Zbtb38 expression alleviates the secondary damages of spinal cord induced by traumatic SCI

**Figure 6:**
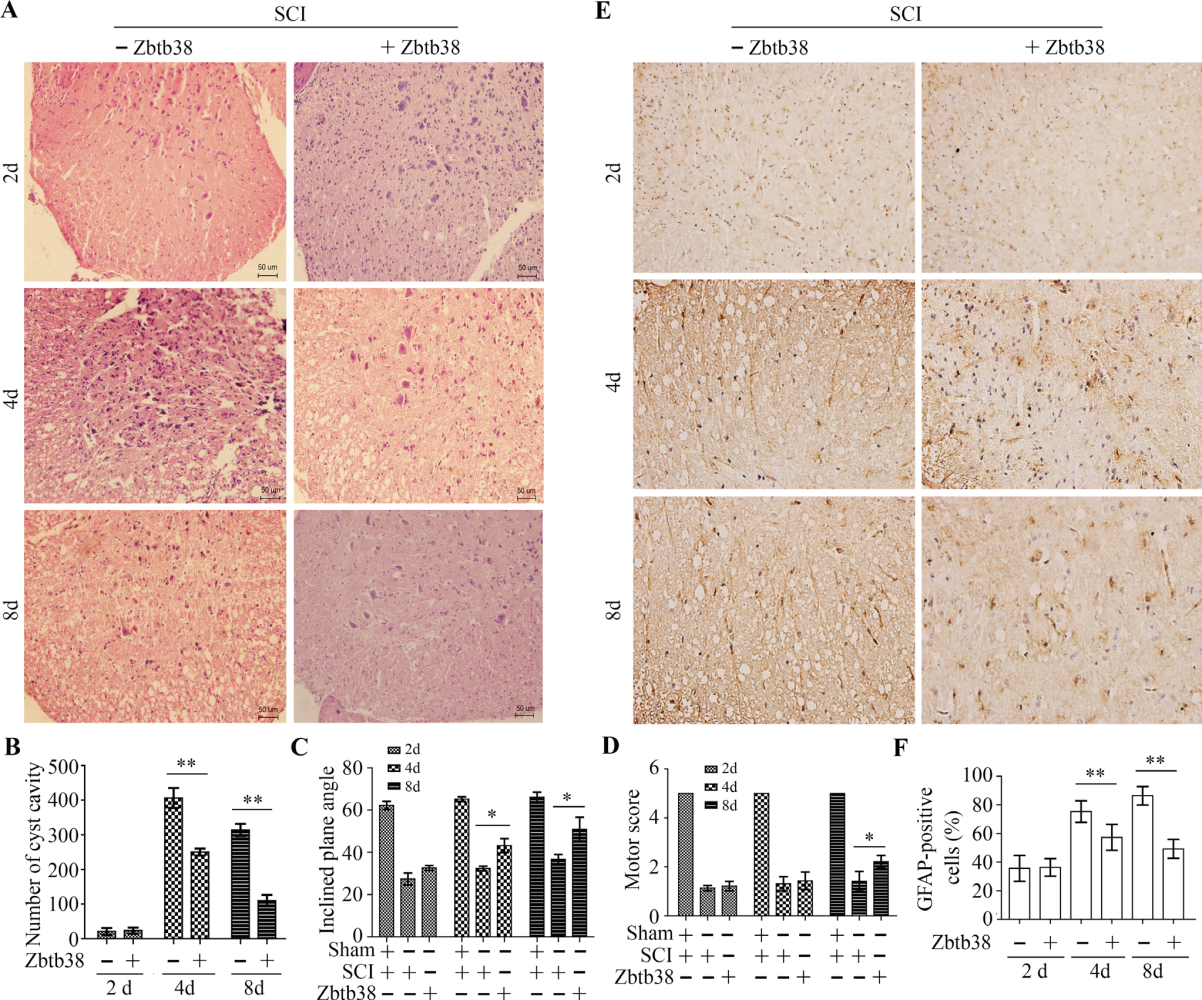
Restoration of Zbtb38 expression alleviates SCI-induced the secondary damages of spinal cord *in vivo* (**A, B**) The spinal cord samples from Zbtb38 lentivirus-treated SCI mice and empty lentivirus-treated SCI mice were used for HE staining. Representative images of these assays are shown in (A) and quantitative data are shown in (B). (**C, D**) The mice from different treatment (*n* = 10) were used for the inclined plate test (C) and motor evaluation (D). (**E, F**) The spinal cord samples from Zbtb38 lentivirus-treated SCI mice and empty lentivirus-treated SCI mice were immunostained with the GFAP antibody. Representative images of these assays are shown in (E) and quantitative data are shown in (F). *p < 0.05; **p < 0.01.

It was possible that restoring Zbtb38 can recover spinal cord functions after SCI. To address it, we performed histopathological analysis, which showed less vacuolization in both gray and white matter in Zbtb38 lentivirus-treated SCI mice compared with that in empty lentivirus-treated SCI mice (Figure 6A and 6B). The inclined plane test showed that the SCI mice performed worse (decreased the inclines plane angle) than control mice on the inclined plane (Figure 6C). There was no difference between Zbtb38 lentivirus-treated SCI mice and empty lentivirus-treated SCI mice on day 2, whereas Zbtb38 lentivirus-treated mice performed much better than empty lentivirus-treated mice on the days 4 and 8 of testing (Figure 6C). Motor test after Zbtb38 lentivirus injection displayed similar tendency to the inclined plane test, showing the scores in Zbtb38 lentivirus treated group were significantly higher than those in empty lentivirus-treated SCI group on 8 day after injection (Figure 6D).

The neural stem cell (NSC)-derived scar component has several beneficial functions, including restricting tissue damage and neural loss after SCI [25, 26]. Accumulation and activation of NSC is essential for self-renewal of injured spinal cord, we thus examined the expression changes of NSC markers NG2 and GFAP in SCI mice, in the presence or absence of Zbtb38 lentivirus. The expression levels of both genes were significantly decreased on the days 4 and 8 after Zbtb38 lentivirus administration (Supplementary Figure 3), suggesting that upregulation of Zbtb38 expression can alleviate SCI-induced secondary damages of spinal cord *in vivo*. Consistently, immunostaining with the GFAP antibody showed that less GFAP positive cells in the injured spinal cord when the Zbtb38 expression was rescued for 4 and 8 days (Figure 6E and 6F). These observations indicate restoring Zbtb38 in injured spinal cord can rescue at least partly the secondary damages of spinal cord.

## DISCUSSION

ER homeostasis is an essential process for the maintenance of cellular homeostasis in CNS, under physiological or pathological conditions [27–30]. Accumulating evidence highlights the importance of ER stress as a common driver of degeneration and neuronal dysfunction after injury to brain, spinal cord and peripheral nerves [31, 32]. However, the mechanisms of ER-associated apoptosis of neurons within injured spinal cord are poorly understood. In this study, we demonstrate that loss of Zbtb38 contributes to secondary damage of traumatic SCI through promoting ER stress-associated apoptosis.

Open-field rating scales are often used to measure locomotor impairments following SCI. The modified Tarlov scale used in this study and the Basso Mouse Scale (BMS) are common locomotor assessment to account for recovery pattern in mice [3]. However, they are designed to assess gross recovery levels across the full range of recovery and lack sensitivity at specific levels of recovery. These disadvantages may in part due to the ordinal nature of the scale. It has been reported that a modified ladder beam instrument and scoring system can be used for assessing hindlimb recovery in vertebral T9 contusion spinal cord injured mice [33]. In future, we will develop more appropriate models to evaluate recovery levels under different conditions.

Our previous study has shown that Zbtb38 negatively regulates apoptosis through mitochondrial pathway after SCI [9]. Here, we confirmed it by new evidence that downregulation of Zbtb38 increased the expression levels of mitochondria-localized pro-apoptotic genes such as Bak, Noxa, Puma, Bim in cultured SH-SY5Y cells and SCI mice. We also found that DR5 gene, encoding a cell surface receptor of the TNF-receptor superfamily to bind TRAIL and mediate apoptosis, was also upregulated when Zbtb38 expression was repressed. These novel findings indicate that Zbtb38 exhibits an anti-apoptotic activity during SCI by blockade of extinct and instinct apoptotic pathways. Although our findings point to a critical role of Zbtb38 in SCI, the mechanistic details such as how Zbtb38 suppresses SCI-induced apoptosis and arrests cell cycle still need to be elucidated.

**Figure 7:**
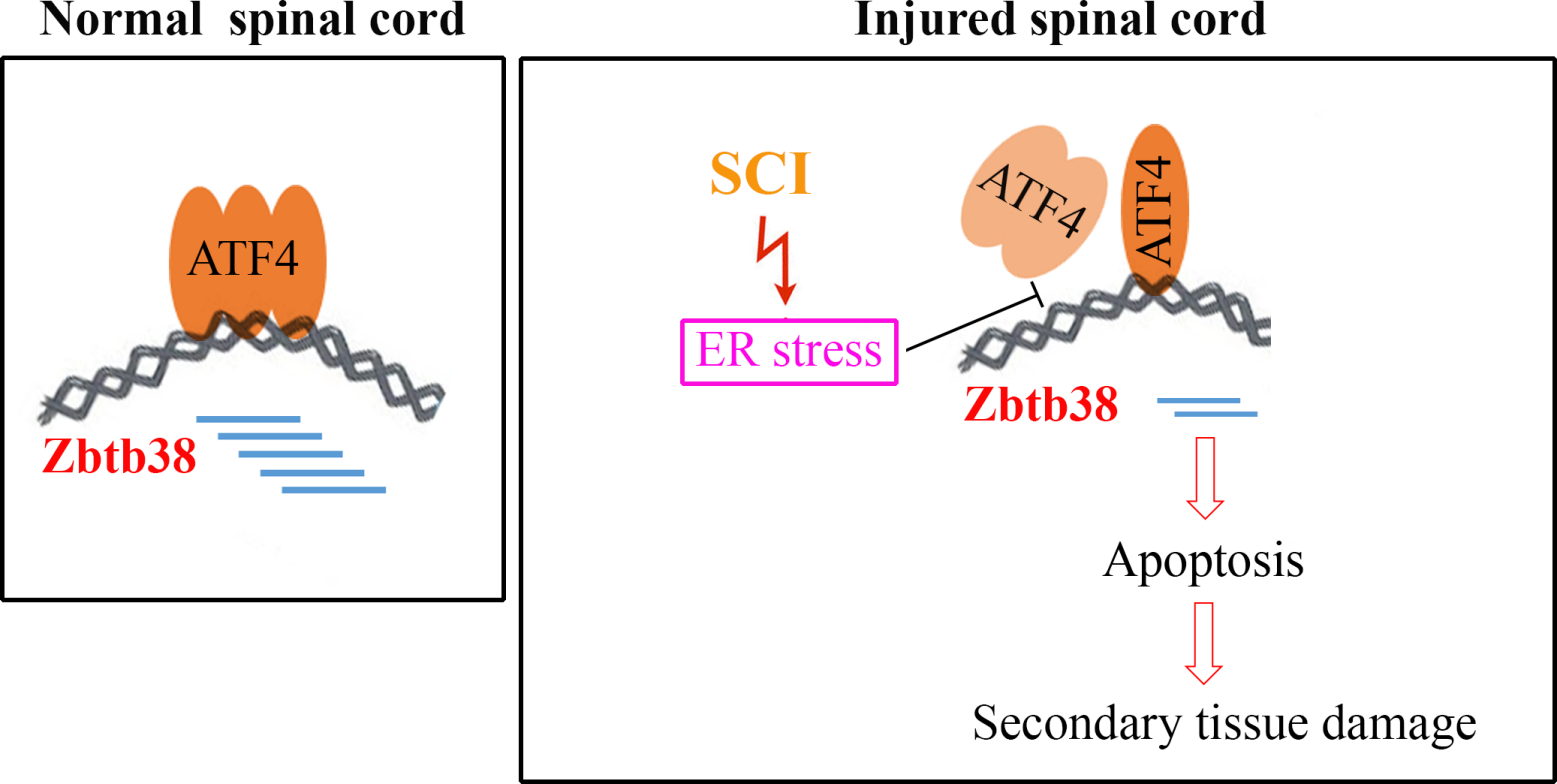
Schematic representation of ATF4-Zbtb38 signaling during SCI

ER stress signaling pathways are partially mediated by ATF4, which in turn activate the downstream targets in response to ER stress [34]. We show, for the first time, that Zbtb38 is a target of ATF4 and its expression is highly controlled by ATF4. During SCI, ER stress is present in spinal cord which blocks ATF4 to bind to the Zbtb38 promoter (Figure 7). As a result, decreased Zbtb38 promotes the secondary damages of spinal cord by loss of its anti-apoptotic function (Figure 7). Therefore, the ER stress-associated ATF4-Zbtb38 cascade is a damage-induced signal in the injured spinal cord. Although there were no significant alteration of ATF4 expression during SCI, reduced amount of ATF4 protein was identified at the promoter of Zbtb38 gene. These data suggest that transcription factors such as ATF4 can transcriptionally regulate target genes under ER stress through altering the binding amount to the targeted DNA promoters.

Additionally, our study also demonstrates that rescue of Zbtb38 can alleviate secondary damage of spinal cord through inhibition of ER stress-associated apoptosis, which provides a novel therapeutic regimen that targeting Zbtb38 may promote functional recovery of spinal cord for patients with SCI. Currently, there is no any commercially available Zbtb38 activator. Therefore, seeking a drug or strategy to upregulate Zbtb38 expression will open up a new avenue for the treatment of SCI patients.

## MATERIALS AND METHODS

### Cell culture and standard assays

SH-SY5Y cells were purchased from American Type Culture Collection (Rockville, MD, USA), and cultured in DMEM medium supplemented with 10% FBS. Transient transfections, lentiviral transduction and infection, Western blot, and flow cytometry analysis were carried out as described previously [35–39].

### qRT-PCR and chromatin immunoprecipitation q-PCR (ChIP-qPCR) assays

For QRT-PCR analysis, total RNA was extracted from cells or mice spinal cord tissues using Trizol (Invitrogen, Carlsbad, CA). cDNA generated by the SuperScript II Reverse Transcriptase kit (Invitrogen) was subjected to QRT-PCR analysis. All amplifications were performed in triplicate with the iCycler iQ System (Bio-Rad) using iQ SYBR Green Supermix (Bio-Rad) according to the manufacturer's instruction. GAPDH (for mouse samples) and β-actin (for human samples) were used as the internal control. For ChIP-qPCR assays, genomic DNA from mice spinal cord tissues was cross-linked using 1% formaldehyde at 37°C for 10 min and then extracted using standard procedures. The DNA was completely sheared by sonication into fragments with a peak around 500 bp as described previously [39, 40], and was subjected to immunoprecipitation with the ATF4 or ATF6 antibody. The purified immune-precipitated chromatin was subjected to qPCR using specific primers. The primers used in QRT-PCR and ChIP-qPCR assays are listed in Supplementary Table 1.

### Constructs, antibodies and reagents

The puromycin-resistant pLKO lentiviral vector containing a short hairpin RNA (shRNA) against Zbtb38 (shZbtb38-1: 5′-ATCACGAGCAGAGGCATATTC-3′; shZbtb38-2: 5′-ATACGGGAGAAAGACGATATC-3′) were used to knock down Zbtb38 gene in SH-SY5Y cells. siRNAs against ATF4 (shATF4-1: 5′-CUGCUU ACGUUGCCAUGAUTT-3′; shATF4-2: 5′-CCCUUCAG AUAAUGAUAGUTT-3′; shATF4-3: 5′-CCUGAAAGA UUUGAUAUAATT-3′) were used to deplete ATF4 gene in SH-SY5Y cells. Scramble shRNA (shNC) and siRNA (siCONT) were used as negative control for knockdown experiment. To construct the Zbtb38 lentiviral expression vector, the full-length human Zbtb38 was cloned into pLVX-Puro vector (Clontech, Mountain View, CA) according to the manufacturer's instructions. Zbtb38 and ATF6 were purchased from NovusBio (Littleton, CO) and Peprotech (Rocky Hill, NJ), respectively. ATF4, GAPDH, eIF2α, phosphor-eIF2α, and cleaved-Caspase3 antibodies were procured from Cell Signaling (Beverly, MA). In Situ Cell Death Detection Kit-POD was purchased from Roche (Basel, Switzerland).

### Traumatic SCI animal model and histopathological analysis

Eight-week old male Kunming mice were purchased from Jiangning Qinglongshan Animal Cultivation Farm (Nanjing, China). All experimental procedures were approved by the Institutional Animal Care and Use Committee (IACUC) at Nanjing Agricultural University. The SCI model was established as described previously [9, 20, 21]. Briefly, the control group underwent sham injuries in which only vertebral plates were cut off without causing any spinal injuries. The spinal processes from Th7-Th9 were exposed and SCI success was confirmed by quick jerks of the hindlimbs observed in trauma-surgery animals. SCI Mice were euthanized by cervical vertebra dislocation at 2, 4 and 8 days after trauma, and spinal cord samples were collected and processed. Spinal cords of Th7-Th9 were fixed in PFA overnight and embedded in paraffin wax. For histological analysis, 7 μm serial sections were stained with hematoxylin and eosin (HE) as we described previously [9]. For immunohistochemistry, the sections were stained with the GFAP antibody (Abcam, Cambridge, MA) as described previously [13].

### Injection of Zbtb38 lentivirus

Mice were divided into 2 groups (*n* = 10) at random after 24 hours of SCI model success: SCI groups were injected with 50 μl empty lentivirus into injury center, and treatment groups were injected 50 μl Zbtb38 lentivirus (titer: 10^8^ TU/ml) into injury center. Spinal cord samples were collected and processed on days 2, 4 and 8 after treatment.

### Behavior observation

Functional recovery of SCI mice was assessed by inclined plane and modified Tarlov scale on days 2, 4, and 8 after injection. The modified Tarlov scale 1–5 was used to grade motor functions: (1) without voluntary hindlimb movements; (2) with tiny voluntary hindlimb movements, cannot stand; (3) can stand but cannot walk; (4) can walk with incoordination of the hindlimbs; (5) can walk normally [9].

### Detection of apoptosis

The Cell Death Detection Elisa kit (Roche, Indianapolis, IN) was used to determine apoptosis by measuring mono and oligonucleosomes in the lysates of apoptotic cells according to the manufacturer's protocol. Apoptotic cells in tissue sections were assessed by TUNEL assay according to manufacturer's instruction. Briefly, the sections were incubated in TUNEL reaction mixture containing TdT and fluorescein-conjugated dUTP to label the ends of DNA fragments. After washing, the labeled preparations were covered using mounting medium containing DAPI for fluorescence (Vector Laboratories, Burlingame, CA). As a negative control, TUNEL enzyme was omitted for some reactions. TUNEL-positive cells were counted from digitally captured images at low magnification using Image Pro software (San Diego, CA). The average number of TUNEL-positive cells was calculated from at least three sections within each region for each animal.

### Statistical analysis

All data were reported as mean ± SD, and analyzed using SPSS version 20.0 (IBM, Armonk, NY, USA). Statistical tests were performed with the Kruskal-Wallis and Mann-Whitney *U-test*. A least significant difference test was used for comparison between groups. *p* < 0.05 was considered statistically significant.

## SUPPLEMENTARY MATERIALS FIGURES AND TABLES




