# Supplementary material for: Zbtb38 is a novel target for spinal cord injury
Source: Oncotarget. 2017 Apr 27;8(28):45356–66. doi: 10.18632/oncotarget.17487 (PMC5542192; doi:10.18632/oncotarget.17487)
Supplement: Supplementary file 1 [file oncotarget-08-45356-s001.pdf]

Zbtb38 is a novel target for spinal cord injury

Supplementary Materials

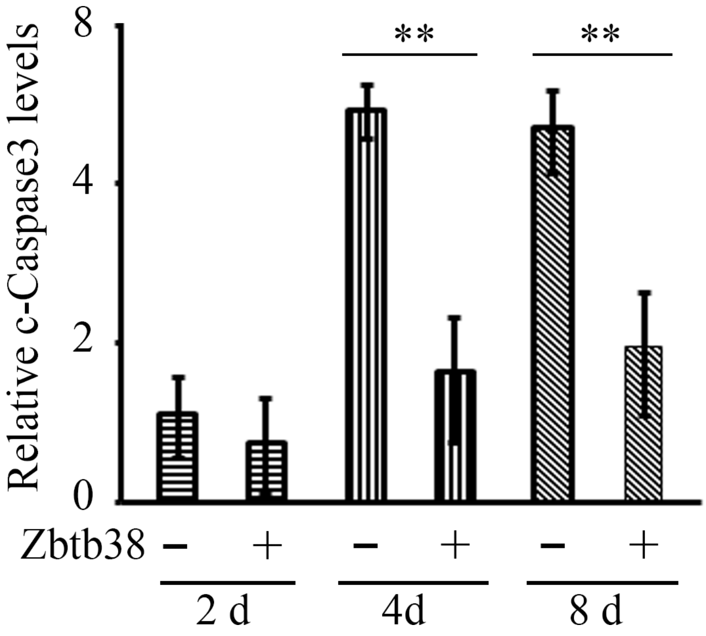

Supplementary Figure 1: Restoration of Zbtb38 expression attenuates SCI-induced Caspase3 cleavage. The tissue lysates were collected from the mice described in Figure 4 for Western blot and quantitative data are shown here. $N = 5$; $^{**}p < 0.01$.

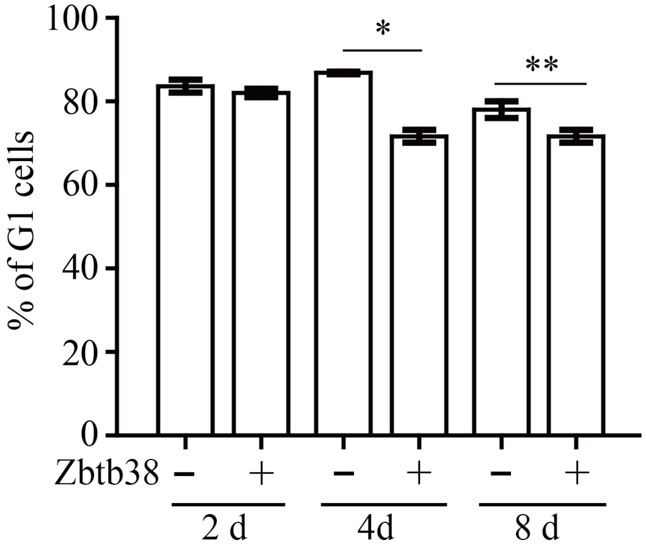

Supplementary Figure 2: Restoration of Zbtb38 expression promotes cell transitions from G1 to S phase after SCI. The cells of spinal cord from the lentivirus-treated SCI mice and non-treated SCI mice were used for flow cytometry analysis after PI staining. Quantitative data of G1 population are shown here. $^{*}p < 0.05$; $^{**}p < 0.01$.

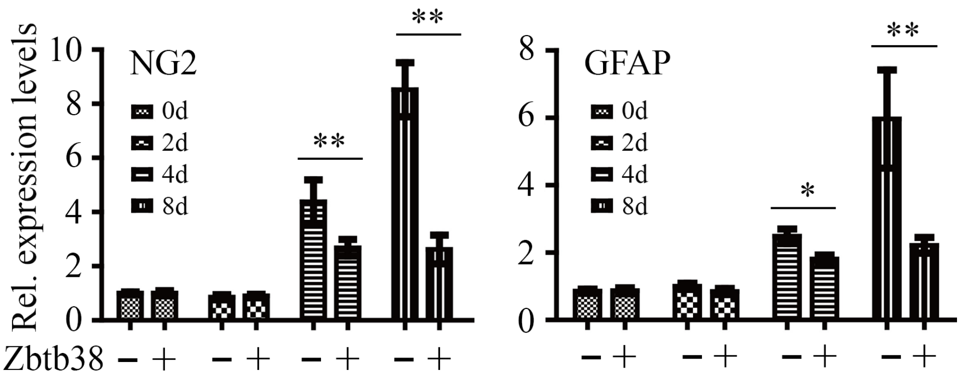

Supplementary Figure 3: Restoration of Zbtb38 expression increases the expression levels of neural stem cell markers after SCI. The RNA from the samples used in Figure 4 was collected to determine the expression levels of neural stem cell markers NG2 and GFAP genes. *$p < 0.05$; **$p < 0.01$.

Supplementary Table 1: The primers used for QRT-PCR and ChIP-qPCR analysis. See Supplementary_Table_1
