# Supplementary material for: Zbtb38 is a novel target for spinal cord injury
Source: Oncotarget. 2017 Apr 27;8(28):45356–66. doi: 10.18632/oncotarget.17487 (PMC5542192; doi:10.18632/oncotarget.17487)
Supplement: Supplementary file 2 [file oncotarget-08-45356-s002.docx]

**Table S1. The primers used for QRT-PCR and ChIP-qPCR analysis**

1. **QRT-PCR**

| **Gene** | **Primer sequences** |
| --- | --- |
| mGapdh | Forward: 5’- AACTTTGGCATTGTGGAAGG 3’  Reverse: 5’- ACACATTGGGGGTAGGAACA -3’ |
| mZbtb38 | Forward: 5’- CCAGAAAATAGGATTGGCGA -3’  Reverse: 5’- GTTGCAGTAATGGCAAGGGT -3’ |
| mChop | Forward: 5’- GCATGAAGGAGAAGGAGCAG -3’  Reverse: 5’- ATGGTGCTGGGTACACTTCC -3’ |
| mERdj4 | Forward: 5’- TAAAAGCCCTGATGCTGAAGC -3’  Reverse: 5’- TCCGACTATTGGCATCCGA -3’ |
| mGrp78 | Forward: 5’- ACTTGGGGACCACCTATTCCT-3’  Reverse: 5’- ATCGCCAATCAGACGCTCC-3’ |
| mBcl-2 | Forward: 5’- ACTGTGTTAACTCCTGCCCG -3’  Reverse: 5’- GCAGCAAGCTACTCAGACGA -3’ |
| mBim | Forward: 5’- TGCAGAGGATGATTGCTGAC -3’  Reverse: 5’- GATCAGCTCGGGCACTTTAG -3’ |
| mPuma | Forward: 5’- GCCCAGCAGCACTTAGAGTC -3’  Reverse: 5’- TGTCGATGCTGCTCTTCTTG -3’ |
| mBak | Forward: 5’- AAAATGGCATCTGGACAAGG -3’  Reverse: 5’- AAGATGCTGTTGGGTTCCAG -3’ |
| mNoxa | Forward: 5’- GGCAGAGCTACCACCTGAGT -3’  Reverse: 5’- TTGAGCACACTCGTCCTTCA -3’ |
| mDR5 | Forward: 5’- TGACTACACCAGCCATTCCA -3’  Reverse: 5’- AGTTCCTCTTCCCCGTCAGT -3’ |
| mGFAP | Forward: 5’- AAGGTTGAATCGCTGGAGGA -3’  Reverse: 5’- ACCACTCCTCTGTCTCTTGC -3’ |
| mNG2 | Forward: 5’- TACAGTGGCTTTGGAGGGAG -3’  Reverse: 5’- GTTGAGAGGCTCCAGTGACT -3’ |
| hβ-actin | Forward: 5’- CCTGTACGCCAACACAGTGC -3’  Reverse: 5’- ATACTCCTGCTTGCTGATCC -3’ |
| hZbtb38 | Forward: 5’- GTCAACTGGAGGAAGGAGCA -3’  Reverse: 5’- GAGAAGTAAGATGTCGGTGGG -3’ |
| hERdj4 | Forward: 5’- TCGGCATCAGAGCGCCAAATCA -3’  Reverse: 5’- ACCACTAGTAAAAGCACTGTGTCCAAG -3’ |
| hGrp78 | Forward: 5’- TTCTTCAATGGCAAGGAACC -3’  Reverse: 5’- TGACACCTCCCACAGTTTCA -3’ |
| hChop | Forward: 5’- CAGAACCAGCAGAGGTCACA -3’  Reverse: 5’- AGCTGTGCCACTTTCCTTTC -3’ |
| hDR5 | Forward: 5’- GCACCACGACCAGAAA -3’  Reverse: 5’- CACCGACCTTGACCAT -3’ |
| hNoxa | Forward: 5’- GCTGGAAGTCGAGTGTGCTA -3’  Reverse: 5’- CCTGAGCAGAAGAGTTTGGA -3’ |

| hPuma | Forward: 5’- ACGACCTCAACGCACAGTACGA -3’  Reverse: 5’- GTAAGGGCAGGAGTCCCATGATGA -3’ |
| --- | --- |
| hBim | Forward: 5’- GCCACTACCACCACTTGAT -3’  Reverse: 5’- CTGGGTCTTGTTGGTTTGA -3’ |
| hBak | Forward: 5’- CTTTCCATGGGTGAGGACAG -3’  Reverse: 5’- AGCTCCCAGGACCTGCACAG -3’ |
| hATF4 | Forward: 5’- CCCCTTCACCTTCTTACAACC -3’  Reverse: 5’- GGGCTCATACAGATGCCACTA -3’ |

1. **For ChIP-qPCR**

| **Gene** | **Primer sequences** |
| --- | --- |
| mATF6 (-1~-500bp) | Forward: 5’- ACTACACAAGGCGGAAGGAA -3’  Reverse: 5’- TGCTTGCTATTTCCAATGGTTG -3’ |
| mATF6 (-500~-800bp) | Forward: 5’- CGCAGCACATGAACAGACTT -3’  Reverse: 5’- GAGGAGGAAGAAGGCACCAT -3’ |
| mATF6 (-800~-1027bp) | Forward: 5’- ATGGTGCCTTCTTCCTCCTC -3’  Reverse: 5’- ACAGAGAAACCCTGTCTCGG -3’ |
| mATF4 (-1~-285bp) | Forward: 5’- GCCTTGTAAGACACCGGAAA -3’  Reverse: 5’- GTGGTCACGTGATCCTACC -3’ |
| mATF4 (-285~-535bp) | Forward: 5’- AGGCTATAAAGGGCGGGTTT -3’  Reverse: 5’- TGCCTCTAATACGCCATGGT -3’ |
| mATF4 (-771~-935bp) | Forward: 5’- GGCCTTGGCCGTATTAGGA -3’  Reverse: 5’- ATTGTGTAAGCCTCCCCGG -3’ |
